# When History Repeats Itself: Exploring the Genetic Architecture of Host-Plant Adaptation in Two Closely Related Lepidopteran Species

**DOI:** 10.1371/journal.pone.0069211

**Published:** 2013-07-12

**Authors:** Hermine Alexandre, Sergine Ponsard, Denis Bourguet, Renaud Vitalis, Philippe Audiot, Sandrine Cros-Arteil, Réjane Streiff

**Affiliations:** 1 INRA, UMR CBGP (INRA, IRD, CIRAD, Montpellier SupAgro), Montferrier-sur-Lez, France; 2 Université de Toulouse, ENFA, UMR5174 EDB (Laboratoire Évolution & Diversité Biologique), Toulouse, France; 3 CNRS, Université Paul Sabatier, UMR5174 EDB, Toulouse, France; Oxford Brookes University, United Kingdom

## Abstract

The genus *Ostrinia* includes two allopatric maize pests across Eurasia, namely the European corn borer (ECB, *O. nubilalis*) and the Asian corn borer (ACB, *O. furnacalis*). A third species, the Adzuki bean borer (ABB, *O. scapulalis*), occurs in sympatry with both the ECB and the ACB. The ABB mostly feeds on native dicots, which probably correspond to the ancestral host plant type for the genus *Ostrinia*. This situation offers the opportunity to characterize the two presumably independent adaptations or preadaptations to maize that occurred in the ECB and ACB. In the present study, we aimed at deciphering the genetic architecture of these two adaptations to maize, a monocot host plant recently introduced into Eurasia. To this end, we performed a genome scan analysis based on 684 AFLP markers in 12 populations of ECB, ACB and ABB. We detected 2 outlier AFLP loci when comparing French populations of the ECB and ABB, and 9 outliers when comparing Chinese populations of the ACB and ABB. These outliers were different in both countries, and we found no evidence of linkage disequilibrium between any two of them. These results suggest that adaptation or preadaptation to maize relies on a different genetic architecture in the ECB and ACB. However, this conclusion must be considered in light of the constraints inherent to genome scan approaches and of the intricate evolution of adaptation and reproductive isolation in the *Ostrinia* spp. complex.

## Introduction

An intriguing yet unresolved question in evolutionary biology is the degree to which evolution is canalized by intrinsic developmental properties [1]. Because we can usually only observe the outcome of evolution and not its path, it is difficult to tell whether independent evolution of the same phenotype reflects historical contingencies or intrinsic constraints [2]. Gould's metaphor [3] to “rewind the tape of life” illustrates this difficulty: what if, starting from the same initial conditions, we could let evolution happen all over again? Could adaptations to a given ecological change have occurred differently from what they actually did?

“Rerunning the tape of life” being… somewhat difficult, an alternative approach is to study naturally occurring cases of repeated adaptations, i.e. adaptations that occurred independently as a response to similar ecological changes. For a long time, evolutionary biologists considered that a trait evolving in closely related lineages (‘parallel evolution’) is likely to rely on the same genetic architecture, whereas a trait evolving in already distant lineages (‘convergent evolution’) is more likely to involve different genetic architectures. However, recent reviews and discussions [1], [4]–[7] suggest that the phylogenetic distance between two taxa is not necessarily a good predictor of the similarity of the genetic architecture of a trait evolving independently in these taxa. Some case studies showed that the same gene can be involved in similar adaptations in phylogenetically distant taxa (e.g., the *MC1R* gene involved in color variation in lizards, birds, felids, mice and black bears [5]). In contrast, some populations within species repeatedly adapted to the same environment through changes in different genes or pathways (see, e.g., the various mechanisms involved in the pelvic reduction in ninespine sticklebacks [8]). With the advent of next-generation sequencing technologies, it is now possible to get insight into the genetic architecture of repeated adaptations, regardless of their occurrence in closely or more distantly related taxa.

The evolution of food preference within the large and diverse group of phytophagous insects is an outstanding source of case studies to analyze repeated adaptations. Phytophagous insects evolve in close interaction with their host-plants, and some authors consider the use of a particular host-plant type as a homoplasic character. For example, in *Nymphalini* butterflies, different species feed on different plant families, and some specific associations have evolved independently several times [9]. Such repeated adaptations are also known in the coleopteran genus *Trirhabda*, in which four independent host-plant shifts from Asteraceae to Anthemideae have been documented [10].

Here, we conducted a comparative analysis of repeated adaptation in phytophagous moths of the genus *Ostrinia* (Lepidoptera, Crambidae). Two *Ostrinia* species have been particularly studied over the last decades because they are major pests of maize (*Zea mays* L.): *Ostrinia nubilalis* Hübner *sensu* [10], the European corn borer (ECB), and *Ostrinia furnacalis* Guénée, the Asian corn borer (ACB). These two species are mostly allopatric at Eurasian scale, the ECB occurring in Europe and the ACB in Asia [12]. A third species, *Ostrinia scapulalis* Walker *sensu* [12], the Adzuki bean borer (ABB), is largely found in sympatry with both maize-feeding species [11], [12]. The major known host plants of the ABB are mugwort (*Artemisia vulgaris* L.: Asteraceae), hop (*Humulus lupulus* L.: Cannabaceae) and hemp (*Cannabis sativa* L.: Cannabaceae), which are all dicots and native to Eurasia, unlike maize.

At least in France, the ECB and ABB do not only differ in host plant use. A large number of behavioral and ecological differences have indeed been documented between these species, most of which can be related either to differences in host plant use and/or to reproductive isolation: oviposition preferences for different host-plants [13]–[15], better larval survival and growth rates on different host-plants [16], contrasted parasitoid communities [17]–[19], local differences in sex pheromone blends produced by females and recognized by males [19]–[21], possibly different sex pheromone blends produced by males and recognized by females [22], slightly shifted phenologies [19], [23], and strong assortative mating [13], [21], [23]. These differences documented in France essentially hold across the former Soviet Union [24]. Although available evidence is scarcer, phenotypic differences related to host plant use and/or reproductive isolation also exist between the ACB and ABB in Asia [25]. Both species are differentiated for oviposition preferences [26]–[28], larval growth and survival rates on maize-like artificial diet [29], female sex pheromones [25], [30] and morphological shape of the male genitalia [12]. Both the ECB and ACB, which have better larval survival and growth rates on maize, as compared to alternative plants, massively infest this crop. Moreover, they are rarely if ever found on the three major ABB host plants, even where the host plants occur in sympatry.

Maize has been domesticated *ca.* 8,700 years ago in the highlands of Mexico. It then spread across the Americas during the next millennia, and was introduced very recently (*ca*. 500 years ago) into Europe [31] and almost simultaneously into Asia [32]. The ability to feed on maize most likely evolved twice, independently in the ECB (in Europe) and the ACB (in Asia) from a common dicot feeding ancestor of the ACB, ECB and ABB (see Figure S1–A). This ability to feed on maize is either the direct consequence of an adaptive shift during maize introduction, or the result of a preadaptation in ECB and ACB. Here, we aimed at determining whether this ability to feed on maize of the ACB and ECB relies on the same or different genetic architecture(s) in both species. By genetic architecture, we mean (in the absence of an assembled reference genome) the number of genomic regions presumably involved in the expression of a trait (namely the ability to use maize as a host-plant) and the extent of linkage disequilibrium between them. In the ECB, adaptation to maize seemingly relies on few independent genomic regions [33]. To determine whether the adaptation of the ACB to maize relies on the same or different genetic architecture(s), we used a ‘genome scan’ approach which has been successfully applied in several organisms to detect signatures of selection on various traits [33]–[42]. We analyzed three pairs of sympatric ECB and ABB populations in France (which were previously screened for divergent selection in [33]), and three pairs of sympatric ACB and ABB populations in China. We expected to find the same (or linked) outlier loci in the ECB-ABB and ACB-ABB comparison if the same evolutionary pathway accounts for adaptation to maize or different (and unlinked) outlier loci if two different pathways account for adaptation to the respective host plants.

Since the *Ostrinia* spp. genome is not sequenced yet, we used non-targeted amplified fragment length polymorphisms (AFLPs). From a total of 684 marker loci, we identified those markers that displayed a pattern of adaptive divergence between pairs of ECB and ABB populations in France, as well as between pairs of ACB and ABB populations in China.

## Materials and Methods

### Ethics statement

No specific permits were required for the described field studies.

### Insect samples

**Figure 1:**
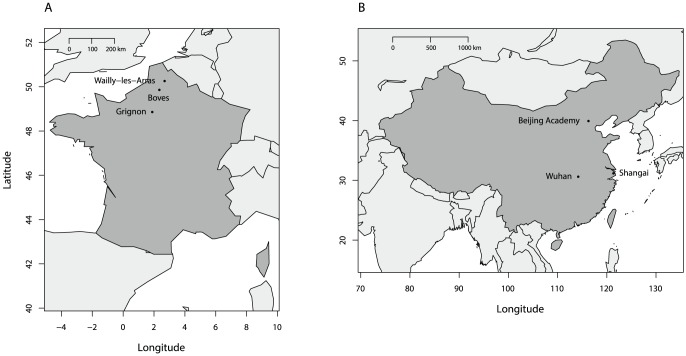
Geographic location of samples in France (A) and China (B).

Diapausing *Ostrinia* spp. larvae were collected from three locations in France (Boves, Grignon and Wailly-les-Arras) and three locations in China (Shanghai, Wuhan and Beijing Academy, Figure 1). In each location, we sampled two populations: one from maize, and one from either hop or mugwort. A total of six pairs of populations were therefore analyzed. Further details are given in Table 1.

**Table 1:** Details on the *Ostrinia* spp. samples collected on maize and dicots in France and China.

Host plant type	Host species	Country	Locality	Population name	Year	*N*	*PL*	% *PL*	*H*_e_ (s.e)
Maize	*Zea mays* L.	France	Boves	BOV-M	2006	24	528	77.2	0.260 (0.007)
			Grignon	GRI-M	2006	24	482	70.5	0.245 (0.006)
			Wailly-les-Arras	WLA-M	2006	24	472	69.0	0.248 (0.007)
		China	Shanghai	SHG-M	2006	21	478	69.9	0.238 (0.007)
			Wuhan	WUH-M	2009	23	501	73.2	0.235 (0.007)
			Beijing Academy	ACA-M	2006	19	470	68.7	0.231 (0.007)
Dicots	*Artemisia vulgaris* L.	France	Boves	BOV-mu	2006	24	486	71.1	0.248 (0.006)
			Wailly-les-Arras	WLA-mu	2006	24	478	69.9	0.249 (0.007)
		China	Wuhan	WUH-mu	2009	21	539	78.8	0.255 (0.006)
	*Humulus lupulus* L.	France	Grignon	GRI-ho	2006	24	455	66.5	0.240 (0.007)
		China	Shanghai	SHG-ho	2006	22	537	78.5	0.252 (0.006)
			Beijing Academy	ACA-ho	2006	21	521	76.2	0.241 (0.006)

*N* is the number of sampled individuals. *PL* and % *PL* indicate the number and percentage of polymorphic loci, respectively. *H*_e_ (s.e.) is the expected heterozygozity and its standard error (s.e.).

### AFLP genotyping

The French samples of the present study have already been analyzed in Midamegbe *et al.* [33]. However, because we used a different molecular ladder in the present study, we could not match with enough confidence the AFLP markers from Midamegbe *et al.*'s [33] study with those from the Chinese populations analyzed here. We therefore produced an entirely new dataset for both Chinese and French populations for the purpose of the present study.

The genomic DNA of each individual was isolated from a piece of the larval body, using either the Qiagen DNEasy Blood and Tissue extraction kit or a standard phenol-chloroform procedure [43]. We then followed the AFLP protocol described in [33] with slight modifications concerning initial DNA amount and PCR characteristics. For each individual, a total of *ca*. 250 ng DNA was digested. The number of cycles (denaturation, annealing, elongation) was increased to 30 for preselective PCRs. The selective PCRs consisted in a 5-min denaturation at 95°C, followed by 13 cycles of a 30-s denaturation at 94°C, a 1-min hybridization starting at 65°C with a 0.7°C decrement per cycle, and a 2-min elongation at 72°C. These 13 cycles were followed by 27 cycles similar to those of preselective PCRs, and by a final 10-min extension at 72°C. To generate AFLP fragments, we used the same set of 13 primer pairs as in [33].

AFLP products were electrophoresed on an ABI 3130XL (Applied Biosystems) sequencer, together with the Internal Lane Standard 600 ladder (Applied Biosystems). Raw data were analyzed with GENEMAPPER© software version 4.0. Samples were first scored for the presence or absence of any given AFLP band (between 80 and 450 bp) on the basis of the automatic procedure in GENEMAPPER©, with intensity threshold set to 20. Then, all samples were visually corrected for, e.g., overlapping bands, unusual peak shape, or dubious peaks. AFLP loci were chosen and scored on the whole data set without considering the origin of the samples (host-plant and country), in order to avoid any ascertainment bias in the definition of the final set of AFLP markers.

### Genetic diversity and population structure

If any two independent AFLP fragments (corresponding to distinct DNA sequences) have exactly the same length, they may be analyzed as a single locus. Since a typical signature for such homoplasy is a negative correlation between fragment size and frequency, we evaluated the level of homoplasy in our dataset by means of the Pearson's correlation test implemented in the software aflp-surv 1.0 [44]. Allele frequencies were estimated using Zhivotovsky's [45] Bayesian method, as implemented in aflp-surv, assuming Hardy-Weinberg equilibrium within each population and for each locus. Nei's [46] gene diversity (*H*_e_) was computed from these allele frequency estimates, and we used Lynch & Milligan's method [47] implemented in aflp-surv to measure genetic differentiation between populations (*F*_ST_).

Clustering analyses based on multilocus genotypes were performed with the Bayesian method implemented in the software package structure [48]. We used version 2.3.1, which handles dominant markers such as AFLPs [49]. We varied *K*, the number of putative clusters, from 1 to 20, and performed 20 independent runs for each value of *K*. We used the admixture model, with the default initial value for *α*, the hyperparameter of the symmetric Dirichlet distribution for the admixture proportions, and assumed that allele frequencies were correlated across populations, using default priors for the *F_k_* parameters. Each Markov chain was run for 500,000 steps, after 50,000 steps of burn-in. We characterized the uppermost hierarchical level of clustering using Evanno *et al.*'s statistic Δ*K* [50], which is based on the rate of change in the log probability of the data between successive values of *K*. We further checked the occurrence of multimodal solutions for a given value of *K* using the *greedy* algorithm implemented in the software clumpp [51].

### Identifying markers involved in host-plant shift and/or in reproductive isolation

We used the software package BayeScan [52] to detect AFLP markers that might be involved (or linked to genes involved) in adaptive divergence between host-affiliated *Ostrinia* species. BayeScan is based on the Multinomial-Dirichlet model for allele frequencies in an island model of population structure. At each locus, the variance of allele frequency between each subpopulation and the common pool of migrants is given by a subpopulation-specific *F*_ST_ parameter. In BayeScan, as in Beaumont & Balding's [53] model, *F*_ST_ is decomposed into a locus-specific component (*α_i_*) shared by all populations, and a population-specific component (*β_j_*) shared by all loci. Positive or negative values of *α_i_* are taken as evidence for selection. BayeScan is based on a reversible-jump Markov chain Monte Carlo (MCMC) algorithm, which provides estimates of the posterior probabilities of two alternative models: one purely neutral (*α_i_* = 0) and one including selection (*α_i_* ≠ 0). For each locus, *Q*-values are computed from the posterior probability that the locus is under selection, allowing for the control of false discovery rate (FDR).

We ran BayeScan version 2.1 (using default parameter values and a minimum allelic frequency equal to 0.05) for all possible pairs of populations, within a country, that involved samples collected from distinct host-plant types (inter-host comparisons) as well as for all possible pairs of populations, within a country, that involved the same host-plant type (intra-host comparisons). Following Nosil *et al.* [37], we considered loci as possible candidates for host-plant adaptation when they were detected as ‘outliers’ – at a given FDR threshold – in at least one inter-host comparison but in none of the intra-host comparisons. This procedure is expected to maximize the chances of detecting selection involved in divergent adaptation to different host-plants, and to minimize those of detecting signatures of selection due to other factors, independent from host-plant adaptation. We examined *α_i_* estimates for all outlier loci, and for each inter-host comparison, as a proxy for the nature and strength of selection: positive *α_i_* values indeed suggest divergent selection while negative values suggest balancing selection.

### Comparison of outlier loci and presumably neutral markers

We performed a hierarchical analysis of molecular variance using the program gda [54], separately for French and Chinese samples. We defined three hierarchical levels: (i) among individuals within population, (ii) among populations within host-plant type and (iii) between host-plant types. For comparison, we performed separate analyses on outliers and on presumably neutral markers (i.e., all markers but the outliers).

### Linkage disequilibrium analyses

We tested for a possible clustering of outlier loci, as different AFLP markers may be located in the same genomic region. To this end, we calculated the level of linkage disequilibrium (LD): (i) within each French population, between pairs of outlier loci detected in French populations; (ii) within each Chinese population, between pairs of outlier loci detected in Chinese populations; (iii) within each population (Chinese or French), between all pairs of loci consisting in one outlier locus detected in French populations and one outlier locus detected in Chinese populations. The latter analyses were aimed at identifying a potential association between the genomic regions under selection in the ACB and ECB. Since AFLPs are dominant markers, we used Hill's [55] eq. (17) to compute the maximum likelihood estimate of the pairwise gametic disequilibrium coefficient *D*_AB_ between any given pair of loci A and B within each population. The pairwise gametic correlation coefficient between pairs of loci was estimated following Dasmahapatra *et al*. [56] as: 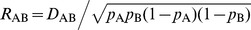, where *p*_A_ and *p*_B_ are the frequencies of the dominant alleles at locus A and locus B, respectively, estimated using Zhivotovsky's [45] Bayesian method.

We developed an original method to test for the significance of gametic correlation coefficients for dominant markers. Hereafter, we refer to the dominant allele (which corresponds to the amplification of one AFLP fragment) by “+” and to the recessive allele (which corresponds to the non-amplification of the AFLP fragment) by “−”. With dominant markers, randomizing the “band absence” and “band presence” phenotypes across individuals at two loci is not an appropriate way of characterizing the distribution of LD under the null hypothesis of linkage equilibrium, since the “band presence” phenotype may correspond either to the “+/+” or to the “+/−” genotype. An appropriate test must therefore be based on the randomization of the underlying genotype frequencies. To this end, we used Zhivotovsky's [45] Bayesian model, but instead of computing the Bayesian estimate of the null-allele (“−”) frequency analytically from his equation (5), we developed an MCMC strategy to sample from the posterior distribution of the null-allele frequency. The rationale was to account for the unknown distribution of allele frequencies, conditionally to the observed distribution of the “band absence” and “band presence” phenotypes, to compute the distribution of LD under the null hypothesis of random association of genes across loci. For each pair of loci, starting from the null-allele (“−”) frequencies calculated from Zhivotovsky's [45] equation (5), each step of the Markov chain consisted in drawing new allele frequencies from a uniform distribution around the current frequencies, within a window of width = 0.15 and with reflecting boundaries at the edges of the interval [0,1]. Each updated allele frequency was accepted according to the appropriate Metropolis ratio derived from the posterior probability given by Zhivotovsky's [50] equation (2). Then, pseudo allele counts of the “+” and “−” alleles were drawn from a binomial distribution, given the allele frequency and the sample size at each locus. Alleles “+” and “−” were randomly assembled as diploid genotypes, and translated into “band absence” and “band presence” phenotypes. The gametic correlation coefficient was computed as above, following Dasmahapatra *et al*. [56], after randomization of the pseudo phenotypes across individuals within a population, for each pair of loci. Each Markov chain was run for 10,000 steps, in order to get the posterior distribution of the pairwise gametic correlation coefficient between pairs of loci under the null hypothesis of random association of genotypes (linkage equilibrium). A *P*-value for each locus pair within each population was then computed as the probability of getting values as large or larger than the one observed.

Finally, LD *P*-values were combined for each of the following categories: populations collected (i) in France on dicots, (ii) in France on maize, (iii) in China on dicots and (iv) in China on maize. To this end, we used the Z-transform method, as advocated by Whitlock [57], implemented in the package survcomp [58] for R [59]. The combined *P*-values were then corrected for the FDR due to multiple testing [60], using the software SGoF+ [61].

## Results

### Population structure

We analyzed 684 AFLP loci in 271 individuals sampled from 12 *Ostrinia* spp. populations (6 pairs of populations). The number of AFLP loci per pair of AFLP primers ranged from 35 to 82, with an average of 53. We did not detect any significant correlation between AFLP fragment size and frequency (Pearson *r* = −0.034, *P* = 0.392).

A total of 558 markers out of 684 were polymorphic using a minimum allele frequency of 0.05 across the whole dataset (i.e., pooling Chinese and French populations). Among them, 92% were shared between French and Chinese samples. Only 1% of the loci were private to pooled French samples and 7% to pooled Chinese samples (private loci were defined as being nearly fixed in one group, i.e. with the “+” allele frequency being larger than 0.99 or smaller than 0.01, but polymorphic in the other group, i.e. with the both allele frequencies comprised between 0.01 and 0.99). Furthermore, most loci were also shared between host-plants within countries, with only 6%, 7%, 5% and 1.5% of the loci being private to French samples on maize, French samples on dicots, Chinese samples on maize and Chinese samples on dicots, respectively. Nei's [46] genetic diversity (*H*_e_) per population ranged from 0.231 to 0.260 with an average of 0.245 and was homogeneous across populations (ANOVA: *F*_11, 259_ = 1.268, *P* = 0.260).

**Figure 2:**
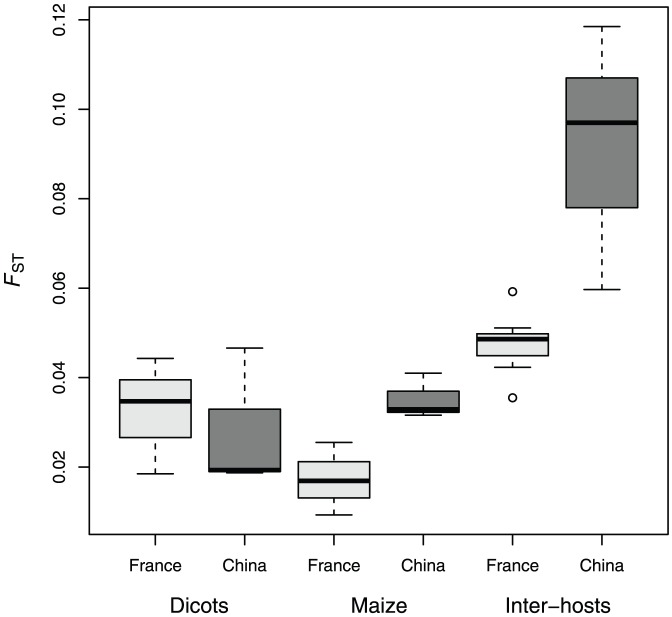
Boxplot of multi-locus *F*_ST_ estimates for pairwise intra- and inter-host comparisons among French (light grey) and Chinese (dark grey) populations.

All pairs of populations were significantly differentiated, regardless of host-plant and country (see pairwise *F*_ST_ estimates provided in Table 2 and represented in Figure 2). The lowest *F*_ST_ values were observed between populations collected on maize in France (*F*_ST_ ranging from 0.009 to 0.026), while the intra-host differentiation was a little higher on dicots in France, on dicots in China and on maize in China (Figure 2). The highest differentiation levels were observed between samples collected on different host-plant types (inter-host differentiation) in China (*F*_ST_ ranging from 0.060 to 0.119) and to a lesser extent in France (*F*_ST_ ranging from 0.036 to 0.059, see Figure 2). The differentiation between Chinese and French populations collected on dicots (inter-countries/intra-host differentiation) ranged from *F*_ST_ = 0.069 to 0.108, and was therefore of the same order of magnitude as the intra-country/inter-host differentiation. As expected, the inter-hosts/inter-countries population pairs were the most differentiated, with *F*_ST_ estimates ranging from 0.056 to 0.179.

**Table 2:** Pairwise *F*_ST_ estimates. Population codes are defined in Table 1.

	BOV-M	GRI-M	WLA-M	BOV-mu	GRI-ho	WLA-mu	SHG-M	WUH-M	ACA-M	SHG-ho	WUH-mu
GRI-M	0.017***										
WLA-M	0.009***	0.026***									
BOV-mu	0.036***	0.050***	0.049***								
GRI-ho	0.051***	0.049***	0.059***	0.044***							
WLA-mu	0.042***	0.045***	0.047***	0.019***	0.035***						
SHG-M	0.132***	0.128***	0.136***	0.145***	0.154***	0.143***					
WUH-M	0.137***	0.137***	0.146***	0.152***	0.165***	0.152***	0.033***				
ACA-M	0.068***	0.151***	0.165 ***	0.170***	0.179***	0.172***	0.041***	0.032***			
SHG-ho	0.068***	0.056 ***	0.071***	0.070***	0.081***	0.069***	0.070***	0.097***	0.108***		
WUH-mu	0.081***	0.084***	0.084***	0.088***	0.108***	0.094***	0.060***	0.097***	0.119***	0.019*	
ACA-ho	0.079***	0.057***	0.087***	0.083***	0.088***	0.080***	0.078***	0.105***	0.107***	0.019***	0.047***

* 0.01<*P<*0.05 and *** *P*<0.001.

**Figure 3:**
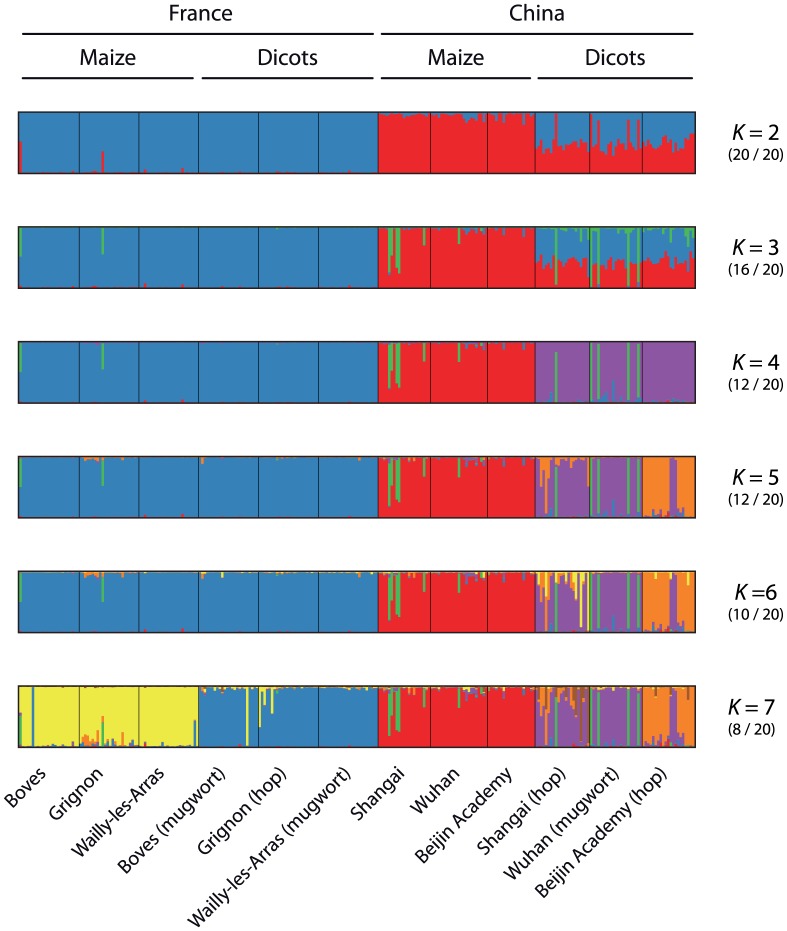
Estimated clustering from structure analyses for *K* = 2 to *K* = 7. Each individual is represented by a vertical line, divided into up to *K* coloured segments representing the individual's estimated fraction of membership of each of the *K* clusters. Vertical black lines separate samples from different localities and host-plants, as labelled at the bottom of the figure. Host-plant and country are indicated at the top. For each value of *K*, the most supported clustering solutions, in terms of number of runs out of 20 (number given to the right of the plot) is represented, whereas alternate solutions are given in Figure S2.

The clustering analysis implemented in structure provided the highest average posterior probability of the data (across 20 runs) for *K* = 7 putative clusters, and the highest value of Evanno *et al.*'s [50] criterion (Δ*K*) for *K* = 2. For *K* = 2, one cluster (in blue, Figure 3) included all French samples collected on maize and dicots, and one cluster (in red, Figure 3) corresponded to samples collected on maize in China. All Chinese samples collected on dicots were admixed, with intermediate membership coefficients in these two clusters. This is consistent with the strong divergence between the ACB and ECB, since Pritchard *et al.* [48] and Fontaine *et al.* [62] showed that the most divergent groups are expected to separate first when increasing the number of clusters. For *K*>2, we observed different clustering results across replicates (Figures 3 and S2), typical of a genuine multimodality [51]. For each value of *K*>2, we found at least one structure solution with a cluster consisting of all French populations (Figures 3 and S2). This pattern is consistent with the stronger differentiation observed among maize- and among dicot-feeding populations in China as compared to France. For *K* ≥ 5, we further found some structure solutions with dicot-feeding individuals clustered in distinct groups (Figures 3 and S2). This suggests that some dicot-feeding populations are differentiated from one another. In contrast, we did not find any evidence of such structure among samples collected on maize neither in France nor in China, regardless of the putative number of clusters.

In general, most individuals sampled from the same population were assigned to the same cluster (Figure 3), although there were two notable exceptions to this pattern: (i) we found some presumably F0 migrants, i.e. individuals that were collected on a given host-plant type, and assigned to the cluster consisting of individuals collected on the other host-plant type in the same location (e.g., in BOV-M and BOV-mu for *K = *7 in Figure 3 and for all values of *K* in Figure S2 except for *K* = 5c and *K* = 7b). These putative migrants were nevertheless kept assigned to the populations they were sampled from, in all subsequent analyses, including BayeScan; (ii) some individuals were assigned to a cluster that was not representative of any of the populations in our data set (green cluster, Figure 3). We compared these results with those obtained using 8 microsatellite loci (data not shown). Four individuals (originating from the WUH-mu Chinese population) showed distinctive genotypes for both the AFLP and the 8 microsatellite loci. Their distinctive genotypes at these independent loci suggested that they might belong to another *Ostrinia* species, different from the ECB, ACB or ABB. These individuals were therefore discarded from the BayeScan analyses. We did not have such clear distinctive patterns for the individuals with mixed assignation probabilities (green cluster/non-green cluster, Figure 3) in BOV-M, GRI-M, SHG-M and SHG-hop, so that these samples were not discarded.

### Identification of markers involved in host-plant shift and/or in reproductive isolation

Following Foll & Gaggiotti's [52] recommendations, we identified outlier loci using different FDR threshold values. In France (ECB vs. ABB comparisons), we found 2 outlier loci at the FDR thresholds 0.05 and 0.10. In China (ACB vs. ABB comparisons), we found 6 outlier loci at the 0.05 FDR threshold and 9 at the 0.10 FDR threshold. These outlier loci correspond to markers that were outliers in at least one inter-host comparison, and in none of the intra-host comparisons. All the outlier loci corresponded to polymorphisms shared between countries (i.e., they were not private, according to the above definition). Yet, none of the outliers detected in France were detected as outliers in China, and vice versa. The *α_i_* estimates for these 11 loci (2 in France and 9 in China) were positive in all within-country inter-host comparisons where they were significantly detected as outliers (Table 3). This suggests that these outliers are under divergent selection between maize- and dicot-feeding *Ostrinia* spp. The average of these *α_i_* estimates ranged from 1.14 to 1.79 for the 9 outliers in China, and equalled 1.60 and 1.69 for the 2 outliers in France.

**Table 3:** Estimates of locus-specific effects *α_i_,* from BayeScan analyses, for each outlier locus in all the inter-host comparisons where it was detected as an outlier (in China and in France).

China										
marker name	SHG-M/SHG-ho	SHG-M/WUH-mu	SHG-M/	WUH-M/SHG-ho	WUH-M/WUH-mu	WUH-M/ACA-ho	ACA-M/	ACA-M/WUH-mu	ACA-M/	
			ACA-ho				SGH-ho		ACA-ho	mean
149								1.35		1.35
156			1.63			1.54				1.59
208				1.65		1.53				1.59
245				1.62	1.95					1.79
306						1.14				1.14
364					1.68	1.39				1.54
401							1.44	1.47		1.46
476					1.64					1.64
606				1.45						1.45

France										
marker name	BOV-M/BOV-mu	BOV-M/GRI-ho	BOV-M/WLA-mu	GRI-M/BOV-mu	GRI-M/GRI-ho	GRI-M/WLA-mu	WLA-M/BOV-mu	WLA-M/GRI-ho	WLA-M/WLA-mu	mean
307	1.65	1.63			1.67		1.74	1.81	1.65	1.69
554			1.58			1.61				1.60

The average of *α_i_* over all these pairwise comparisons is also provided. These values are a proxy for the nature and strength of selection: positive *α_i_* values suggest divergent selection while negative values suggest balancing selection. Population codes are defined in Table? 1. Marker names are underlined (not underlined) for outliers detected at a 5% FDR (10%) threshold.

Among French samples, the *F*_ST_ estimates were 0.704 for the French outliers, and 0.045 for the presumably neutral markers; among Chinese samples, the *F*_ST_ estimates for the Chinese set of 6 (resp. 9) outliers were 0.678 (resp. 0.631), and 0.102 (resp. 0.098) for the presumably neutral markers. Both in France and China, the largest component of genetic variance was between host-plant types for outlier loci, and between individuals within populations for presumably neutral loci (see Table 4).

**Table 4:** AMOVA results for outlier and neutral markers, with FDR fixed at 5 and 10%, in French and Chinese populations.

	France (FDR 5% and 10%)				China (FDR 5%)				China (FDR 10%)			
	outliers (2)		neutral (682)		outliers (6)		neutral (678)		outliers (9)		neutral (675)	
Source of variation	variance components	coancestry coefficients	variance components	coancestry coefficients	variance components	coancestry coefficients	variance components	coancestry coefficients	variance components	coancestry coefficients	variance components	coancestry coefficients
Between host-types	1.092	0.704 (0.489–0.869)	8.501	0.045 (0.036–0.054)	3.072	0.678 (0.521–0.803)	20.937	0.102 (0.085–0.119)	4.147	0.631 (0.507–0.747)	19.861	0.098 (0.081–0.114)
Among populations within host-types	0.032	0.720 (0.528–0.866)	8.970	0.092 (0.083–0.102)	0.153	0.712 (0.599–0.806)	8.267	0.142 (0.126–0.159)	0.282	0.674 (0.585–0.763)	8.137	0.138 (0.122–0.154)
Among individuals within populations	0.435		172.185		1.305		175.996		2.142		175.158	

Coancestry coefficients are computed according to [54].

We did not find any significant LD (FDR-corrected *P*-values>0.05), neither between the 2 outliers detected in France, nor between the 36 possible pairs of outliers detected in China. Likewise, we did not find any significant LD between the 18 possible pairs consisting of one outlier detected in France and one detected in China.

## Discussion

Comparative analyses of independent occurrences of similar adaptations in closely related taxa can help in disentangling the role of historical contingencies from that of intrinsic constraints in evolution. The genus *Ostrinia* offers an outstanding situation, because the ability to feed on maize most likely evolved twice independently in the ECB (in Europe) and the ACB (in Asia) (see Figure S1). Whether this ability evolved on maize itself during the colonization of this new host plant, or prior to its introduction into Eurasia (e.g., as an adaptation to another, yet unknown, maize-like host plant) is, to date, unresolved. Indeed, the divergence between the studied taxa most likely preceded the introduction of maize [63], [64], which suggests that the genetic changes involved in the initial divergence may have been caused by local adaptation on other host plants. Hence, what we call ‘adaptation’ or ‘preadaptation’ to maize is the evolution of one or several traits that, compared to the ABB, increase the performance of the ACB and ECB when feeding on maize and/or reduce their performance when developing on the major host plants of the ABB (mugwort, hemp and hop). Therefore, we do not make any assumption as to the timing or the cause of this adaptation in the present study.

### A small number of outlier loci

We detected a small proportion of the genome to be involved in the adaptive divergence between the dicot-feeding and the maize-feeding taxa both in France and in China. This result is in line with those compiled by Matsubayashi *et al.* [65], who found that the genetic architecture of host preference and performance (two major traits involved in host-plant adaptation) can involve few loci with major effect in various species and orders of phytophagous insects, including Lepidoptera. While Matsubayashi *et al.*'s review referred to quantitative genetics studies, recent molecular studies on host shifts in *Drosophila* and aphids carried on this characterization by identifying two major classes of gene *functions* underlying host plant adaptation. The first class refer to chemoreceptor genes such as odorant, gustatory receptor genes and genes encoding salivary proteins in the pea aphid, *Acyrthosiphon pisum* [66], [67] or odorant binding protein in *Drosophila sechellia* [68]. The second class of functions encompasses genes involved in detoxification and metabolism, as shown by population transcriptomics in *D. mojavensis* [69] and *D. sechellia* [68]. In *Ostrinia*, the association between AFLP outliers detected in our study and candidate genes remains to be established. Yet, chemoreceptor genes would appear as relevant candidates since a recent flight tunnel study showed that ECB adults discriminate volatile organic compounds emitted by host plants [70]. In the larval stage, UDP-glucosyltransferase or other UDP-glucose-dependent enzymes might also be good candidates for host plant adaptation since these enzymes are probably involved in the capacity of the ACB to feed on plants, like maize, that contain cyclic hydroxamic acids (cHx) [29].

### Different genomic regions under selection in the ACB and ECB?

We found that different and unlinked outliers differentiated ECB from ABB in France, and ACB from ABB in China. Detecting different genomic regions under selection in the ACB and ECB has two major implications. First, it strengthens the hypothesis of two independent adaptation events to maize. Indeed, if there had been only one adaptation event to a maize-like plant followed by a reversion to the ancestral host plant(s) in the ABB (see scenario B in Figure S1), we would expect genes involved in adaptation to be the same in the ECB and ACB, and to find either the same or different, yet linked, outlier loci in the ECB and ACB. Second, it suggests that host plant adaptation in the ECB and ACB have different genetic architectures, and that the ancestral species of the ABB, ACB, and ECB had at least two (and possibly more) potential ways of becoming adapted to maize. Although preliminary, this result also opens a series of questions about the underlying mechanisms. If truly independent, did mutations arise on different genes belonging to the same metabolic pathway, or through different mutations/rearrangements of the same target genes? Do the genetic changes conferring adaptation correspond to regulatory or structural mutations? Did adaptation involve duplication events followed by neofunctionalization on different copies in both species? However, alternative interpretations of these results must also be considered, as detailed below.

### The limits of genome-scan approaches

#### Stringency of the statistical methods

As compared with other genome scan studies (which typically report 5–10% of outliers: see [71]), the proportion of outlier loci found in the present study is rather low, both in France (0.29% of outlier loci in ABB vs. ECB comparisons) and in China (0.9% or 1.3% in ABB vs. ACB comparisons, depending on the FDR threshold). In particular, we found fewer outlier loci than in a previous analysis of the same ABB and ECB samples in France (2.3% of outliers using a different set of AFLP markers, see: [33]). One reason for this difference could be that our approach was more stringent in two respects. First, we used BayeScan [52] in the present study, which has been shown to provide a lower false-positive rate (at least in certain conditions, see [72]) than the methods used in Midamegbe *et al.* [33]: DetSel [73]–[75] and Fdist2/Dfdist [76]. Second, we used the FDR correction for multiple testing implemented in BayeScan 2.1, which was not applied in Midamegbe *et al.* [33]. Applying the same methods as in Midamegbe *et al.* [33] to the French dataset of the present study, we found similar proportions of outliers in both studies (see Table S1). This suggests that the more conservative approach adopted in the present study accounts, at least in part, for the lower number of outliers found here, as compared to Midamegbe *et al.*'s [33] study, and perhaps more generally to the studies reported in Nosil *et al.* [71].

Finally, our strategy consisted in comparing all possible pairs of populations involving samples collected from distinct host-plant types within one country. However, replicates of inter-host comparison cannot be truly considered as independent. Indeed, since individuals are clustered by host-plant type rather than by geography, based on genome-wide data (see Figure 3), these replicates involve populations that share a common history and/or are connected by gene flow. Therefore, we refrained from giving more weight on outliers detected in multiple inter-host comparisons. On the opposite, we were very stringent in discarding all the outliers detected in any of the intra-host comparisons. An alternative would have been to compare the pools of all individuals collected on the same host within one country, regardless of population. The limited sample size in each pairwise test is likely to decrease the power of our analyses. However, ignoring the hierarchical structure of the data (geographical structure within host-plant affiliated species) may lead to a high number of false positives [77]. Unfortunately, no method accounting for hierarchical structure is available to date for biallelic dominant markers such as AFLPs (but see [77], [78] for codominant markers).

#### Limited genome coverage

Hitchhiking reduces variability and enhances differentiation over regions linked to genes under divergent selection, leading to genome-wide variation in the course of divergence, which has been referred to as the “genetic mosaic of speciation” [79]. In non-model organisms, before the advent of next-generation sequencing technologies, most genome scans have been, and often still are, performed with hundreds to thousands of markers, including AFLPs [33], [38], [40], [80], [81]. In *Ostrinia* spp., the genome size is estimated to be *ca*. 500 Mb [82], covering a haploid number of 31 chromosomes [83]. Under the assumption that the 684 AFLP markers used here are randomly distributed across the genome, we therefore expect that, on average, our markers are separated by *ca*. 730 Kb. While some studies support the notion that adaptive divergence results from selection acting on small and localized genomic regions (see [84], [85] and [86] for an empirical illustration in *Heliconius* butterflies), others tend to show that genetic signatures of selection can spread over long genomic regions (e.g., in aphids [87], lake whitefish [88] and sticklebacks [89]). Since the limited genome coverage of the present study may have impeded the detection of localized and/or weakly selected regions, we acknowledge that the low number of outliers found here may only represent a small fraction of the genes actually involved in host plant adaptation.

#### Different levels of shared polymorphism

A third reason why the number of outliers found in the present study might not accurately reflect the number of genes under selection has recently been suggested by Via [87]. Her argument is that, although reduced recombination around a gene targeted by divergent selection may very well spread over genomic blocks as large as several megabases, not every marker locus within a block is expected to be an *F*_ST_-outlier. Non *F*_ST_-outliers within these blocks may indeed correspond to ancestral polymorphism that has not yet significantly diverged in spite of hitchhiking. If Via's [87] argument holds, we would expect the proportion of detectable outliers to depend on the level of shared ancestral polymorphism. In *Ostrinia* spp., previous studies have shown that the ABB and ECB diverged recently [64] – more recently than the ABB and ACB [63], [90]. This implies that the ABB and ECB may share more ancestral polymorphism than the ABB and ACB. In this case, for a given number of loci under divergent selection, we would expect the number of detectable outliers to be smaller in the former than in the latter comparison, although it is difficult to quantify the magnitude of the difference to be expected. Hence, the lower number of outliers in the ECB/ABB as compared to the ACB/ABB comparison (2 versus 9 outliers at the 0.10 FDR threshold) may reveal a genuinely smaller number of loci under selection, but may also result from the higher shared ancestral polymorphism in the ECB/ABB.

**Figure 4:**
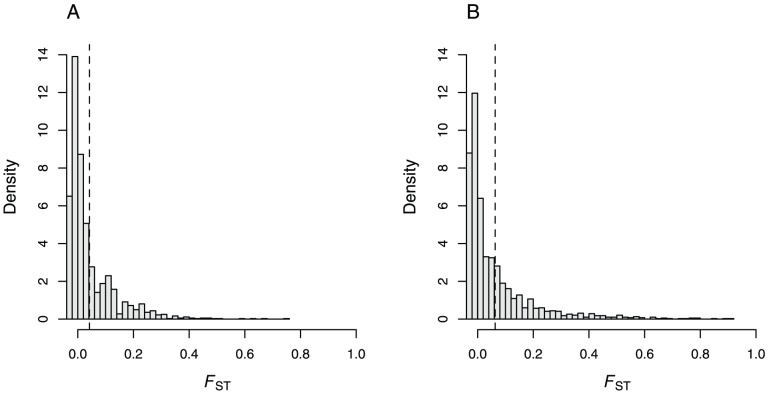
Distributions of *F*_ST_ estimates between populations sampled on different host plants, across all AFLP markers in France (A, including all pairs of ECB and ABB populations) and in China (B, including all pairs of ACB and ABB populations). Mean values of the distribution are 0.042 and 0.063, respectively, as indicated by the vertical dashed lines. Both distributions are highly leptokurtic (i.e. with kurtosis>3) and significantly different from one another (Kolmogorov-Smirnov test, *D* = 0.089, *P*<10^−5^). Higher kurtosis is observed for the ECB/ABB *F*_ST_ distribution (kurtosis = 12.16) than for the ACB/ABB *F*_ST_ distribution (kurtosis = 11.46).

Our results further suggest that the genome-wide variability in divergence differs between species pairs, as illustrated by the distribution of *F*_ST_ estimates (Figure 4). The smaller variance and higher kurtosis of the ABB vs. ECB *F*_ST_ distribution as compared to the ABB vs. ACB *F*_ST_ distribution may result from the more recent divergence between the former pair of species. In the earliest stages of divergence, we expect only a limited number of localized regions of the genome to be differentiated, with most of the genomic differentiation distributed around the mean (see, e.g., Figure l in [91]). This would result in a skewed distribution such as that observed in Figure 4 for the ECB vs. ABB comparison. In later stages of divergence, we expect differentiation to spread around the initial localized regions, and eventually to form islands of isolation, which would result in a more scattered distribution, like the one observed in Figure 4 for the ACB vs. ABB comparison. Both the higher ancestral polymorphism shared by the ECB and ABB and their narrower regions of divergence may therefore account for the lower number of outliers detected in this species pair. Importantly, this pattern is expected regardless of the fact that the genes targeted by selection for the adaptation to maize are the same in the ECB and ACB.

#### Outliers may be related to reproductive isolation rather than adaptation to host plant

We have implicitly assumed so far that adaptation to maize or to an intermediate “maize-like” host plant was the main force driving differentiation between species in each pair. We also implicitly assumed that this adaptation has occurred earlier in the ACB than in the ECB, as suggested (but not demonstrated) by the fact that the ACB and ABB are more differentiated than the ECB and ABB. However, more complex scenarios are possible. For example, in one or both species pairs, divergence may have been initially triggered – and possibly driven for some time – by other factors than host-plant use. If so, adaptation to a new host could have occurred much later. We must therefore keep in mind that, as pointed out by Bierne *et al.* [92], *F*_ST_-outliers found in genome scans may result from reproductive isolation rather than from host plant adaptation. In fact, outliers can be involved both in reproductive isolation and in host plant adaptation if ECB and/or ACB diverged trough ecological speciation events [93]. Indeed, in such kind of speciation, divergent selection for host plant adaptation may directly cause reproductive isolation – e.g., genetic differences in the time of emergence timing that ensure a good match with host plant phenology can in turn ensure strong assortative mating. There are numerous candidate mechanisms for reproductive isolation in *Ostrinia* spp. Roelofs *et al.* [94] conjectured a plausible scenario of divergence between the ACB and the common ancestor of the ECB and ABB: their scenario assumes that the evolution of a different female sex pheromone in the ACB, coupled with a pre-existing polymorphism in male reception system, enabled certain males to detect and respond to the new pheromone. Differences in sexual communication signals by means of ultrasounds [95] may also be involved. Female pheromone polymorphism also exists within the ECB/ABB species pair, although it is present both within the ECB and within the ABB [20], [25], [30], [82], [96], which challenges its possible contribution to reproductive isolation between the ECB and ABB [21]. Other hypothetical contributors to reproductive isolation include a still elusive “assortative mating” (*Am*) trait [21], a male pheromone distinct from the female one [22] and temporal isolation [96]–[98]. Although these traits are unlikely to be directly involved in host plant adaptation, their evolution may have been triggered by reinforcement.

If genome scans were exhaustive, we should therefore detect loci involved in reproductive isolation together with loci involved in host-plant adaptation. However, there is no *a priori* reason to expect the former to be identical in both species pairs. Hence, shared outliers in both species pairs are better candidates for adaptation to the host-plant than to reproductive isolation – unless reproductive isolation and adaptation to host plant are ensured by the same or tightly linked loci/genomic regions (see above). As genome scans are not exhaustive though, and since none of the outliers we detected was shared between the two pairs of species, we cannot exclude that most, if not all, outlier loci are involved in reproductive isolation.

### Conclusion and perspectives

Overall, our results suggest that adaptation to maize has a different genetic architecture in the ACB and ECB. Sequencing the outliers between the ECB and ABB identified in Midamegbe *et al.* [33], and the new set of outliers identified here, will therefore offer the opportunity to perform a comparative analysis between these two taxa. Analyzing new populations and/or increasing genome coverage in future genome scan studies may also help refining the genetic architecture underlying host-plant adaptation by revealing additional outlier loci and ruling out false positives. Mapping outlier loci along a genome draft could complement our approach based on linkage disequilibrium measures by telling whether some sets of outlier loci are clustered in localized genomic regions. Moreover, such an approach could help identifying candidate genes, which would inform us on the enzymes or other proteins involved in adaptation to maize. Comparing the divergence of these candidate genes with non-coding regions in different *Ostrinia* spp. may also help deciphering the putative historical scenarios (Figure S1), and lay the foundations for a better understanding of the evolutionary history of the repeated encounters between maize and its pests-to-be in Asia and Europe.

## Supporting Information

Figure S1 Alternative scenarios for the evolution of adaptation or preadaptation to maize in the ECB and ACB, starting from an ancestral species common to the ACB, ECB and ABB.
(PDF)

Figure S2 Estimated clustering from structure analyses for *K* = 2 to *K* = 7. Each individual is represented by a vertical line, divided into up to *K* coloured segments representing the individual's estimated fraction of membership of each of the *K* clusters. Vertical black lines separate samples from different localities and host-plants, as labelled at the bottom of the figure. Host-plant and country are indicated at the top. For each value of *K*, the less supported clustering solutions, in terms of number of runs out of 20 (number given to the right of the plot) are represented, whereas the most supported solution is given in Figure 3.
(EPS)

Table S1 Number of outlier markers detected using methods alternative to BayeScan.
(DOC)
