# Supplementary material for: When History Repeats Itself: Exploring the Genetic Architecture of Host-Plant Adaptation in Two Closely Related Lepidopteran Species
Source: PLoS One. 2013 Jul 12;8(7):e69211. doi: 10.1371/journal.pone.0069211 (PMC3709918; doi:10.1371/journal.pone.0069211)
Supplement: Figure S1 — Alternative scenarios for the evolution of adaptation or preadaptation to maize in the ECB and ACB, starting from an ancestral species common to the ACB, ECB and ABB. (PDF) [file pone.0069211.s001.pdf]

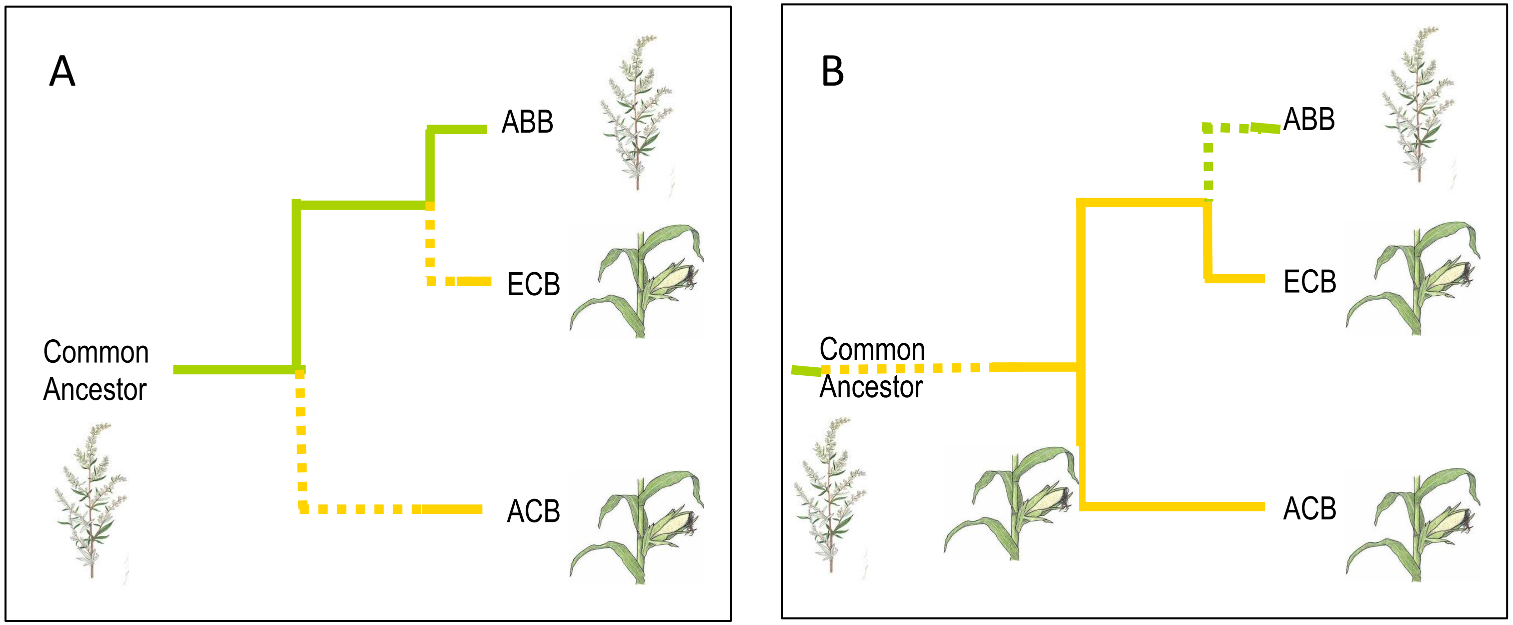

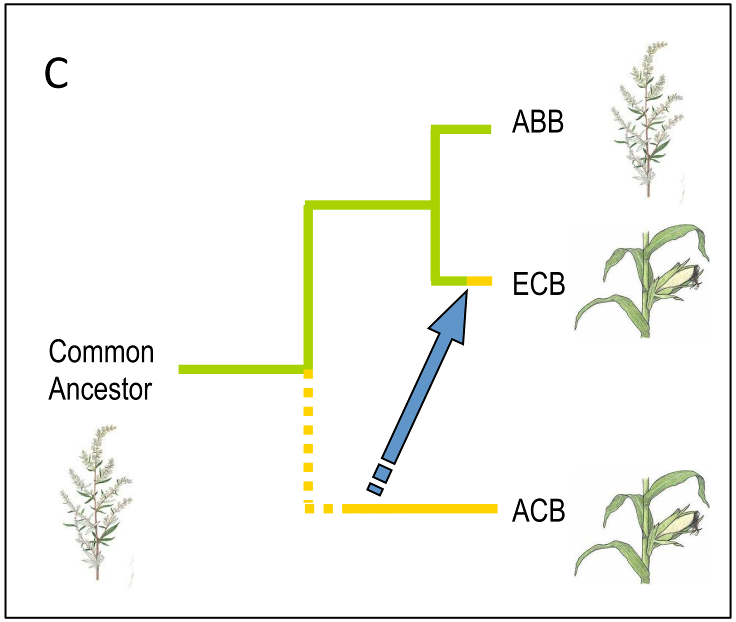

Figure S1 : Alternative scenarios for the evolution of (pre)adaptation to maize in the ECB and ACB, starting from an ancestral species common to the ACB, ECB and ABB. The phylogenetic topology between ECB, ABB and ACB arises from Ishikawa *et al.* 1999. Only one of the major host plants of the three *Ostrinia* species are represented: maize for the ECB and ACB, mugwort (as a proxy for a variety of native dicots) for the ABB and (according to the phagy in the genus *Ostrinia*) for the common ancestor of the three species. The genetic change(s) corresponding to a (pre)adaptation to maize or dicots may have occurred at any time in the dotted parts of the orange or green branches, respectively. (to be continued, page 2)

Figure S1 (continued):

In scenario (A), the ECB and ACB underwent two independent genetic changes allowing them to feed on maize. These changes may have occurred prior to or after introduction of maize into their range, and may or may not have triggered the speciation events between ECB and ABB and/or between the ACB and the ECB-ABB group. In scenario (B), the common ancestor shifted from dicots to (an)other unknown host plant(s), which later facilitated the colonization of maize by the ACB and ECB, as they were thereby “pre-adapted” to feed on maize. A second host change occurred in the ABB, which reversed to feeding on native dicots. Again, both adaptation events may or may not have triggered speciation events.

In scenario (C), the adaptation to maize (or to one or several maize-like host(s)) was acquired either by the ACB or by the ECB and then introgressed into the other species. The picture shows an adaptation in the ACB prior to an introgression into the ECB, but the reverse could also be imagined. Again, the (pre)adaptation to maize may or may not have triggered speciation.

Based on the minimal number of mutations or introgressions, none of the three scenarios is clearly more parsimonious than the other two. However, the geographical distribution of the three species makes scenario (A) (two independent adaptations to maize in the ECB and ACB) more likely than the other two. Indeed, in scenario (B), the ABB would have diverged from the ECB by reversion to feeding on dicot. The occurrence of the ABB over all Northern Eurasia while the ECB is restricted to Western Eurasia favors scenario (A) over scenario (B). Similarly, the fact that the ACB and ECB are almost completely allopatric whereas they are both largely sympatric with the ABB makes scenario (C) somewhat unlikely : why would (pre)adaptation to maize have been introgressed from the ACB into the ECB (or the reverse) but not into the ABB ? There is no definitive evidence in favor of scenario (A), but it appears as the most likely among the most parsimonious scenarios available.

Ishikawa, Y., T. Takanashi, et al. (1999). "*Ostrinia* spp. in Japan: their host plants and sex pheromones." *Entomologia Experimentalis et Applicata* 91(1): 237-244.
