# Supplementary material for: When History Repeats Itself: Exploring the Genetic Architecture of Host-Plant Adaptation in Two Closely Related Lepidopteran Species
Source: PLoS One. 2013 Jul 12;8(7):e69211. doi: 10.1371/journal.pone.0069211 (PMC3709918; doi:10.1371/journal.pone.0069211)
Supplement: Table S1 — Number of outlier markers detected using methods alternative to BayeScan. (DOC) [file pone.0069211.s003.doc]

**Table S1** Number of outlier markers in the present dataset detected in inter-host comparisons from French populations, using the same statistical procedures as in Midamegbe *et al.* for three critical values (0.01, 0.05, 0.10). The number of outliers found in Midamegbe *et al.* is provided between brackets. Midamegbe *et al.* analyzed the same samples in France, but used a different AFLP dataset.

|  | Population pair | |  | Number of outlier loci (Midamegbe *et al.* [40]) | | |
| --- | --- | --- | --- | --- | --- | --- |
| Analysis | Locality (host) | Locality-host | Among / within host | *P* ≤ 0.01 | 0.01 < *P* ≤ 0.05 | 0.05 < *P* ≤ 0.10 |
| DetSel | BOV-mu | BOV-M | Among | 13 (14) | 39 (33) | 35 (38) |
|  | GRI-ho | GRI-M | Among | 11 (15) | 37 (35) | 34 (35) |
|  | WLA-mu | WLA-M | Among | 16 (13) | 30 (47) | 46 (43) |
|  | WLA-mu | BOV-mu | Within | 12 (12) | 37 (34) | 41 (48) |
|  | WLA-mu | GRI-ho | Within | 13 (22) | 39 (36) | 50 (36) |
|  | GRI-ho | BOV-mu | Within | 6 (10) | 34 (32) | 42 (39) |
| DFdist | BOV-mu | BOV-M | Among | 15 (21) | 33 (43) | 36 (39) |
|  | GRI-ho | GRI-M | Among | 22 (25) | 27 (39) | 27 (30) |
|  | WLA-mu | WLA-M | Among | 25 (17) | 26 (41) | 24 (36) |
|  | WLA-mu | GRI-ho | Within | 15 (23) | 28 (37) | 31 (25) |
|  | GRI-ho | BOV-mu | Within | 14 (17) | 21 (27) | 31 (39) |

1. Midamegbe A, Vitalis R, Malausa T, Delava E, Cros-Arteil S, et al. (2011) Scanning the European corn borer *(Ostrin*ia spp.) genome for adaptive divergence between host-affiliated sibling species. Molecular Ecology 20: 1414-1430.
